# The effect of menthol rinsing before intermittent exercise on physiological, physical, and thermo-behavioral responses of male football referees in hot and humid environment

**DOI:** 10.3389/fspor.2024.1334739

**Published:** 2024-01-22

**Authors:** Maria Roriz, João Brito, Filipe J. Teixeira, Konstantinos Spyrou, Vitor Hugo Teixeira

**Affiliations:** ^1^ Faculty of Nutrition and Food Sciences, University of Porto (FCNAUP), Porto, Portugal; ^2^ Portugal Football School, Portuguese Football Federation, Oeiras, Portugal; ^3^ Interdisciplinary Center for the Study of Human Performance (CIPER), Faculdade de Motricidade Humana, Universidade de Lisboa, Cruz-Quebrada, Portugal; ^4^ Atlântica, Instituto Universitário, Fábrica da Pólvora de Barcarena, Barcarena, Portugal; ^5^ UCAM Research Center for High Performance Sport, UCAM Universidad Católica de Murcia, Murcia, Spain; ^6^ Facultad de Deporte, UCAM Universidad Católica de Murcia, Murcia, Spain; ^7^ Research Centre in Physical Activity, Health and Leisure (CIAFEL), Faculty of Sports, University of Porto (FADEUP), Porto, Portugal; ^8^ Laboratory for Integrative and Translational Research in Population Health (ITR), Porto, Portugal

**Keywords:** cooling, football referees, heat stress, intermittent exercise, oral menthol, thermal perception

## Abstract

**Introduction:**

In the current experiment, we aimed to evaluate whether eliciting pre-exercise non-thermal cooling sensations would alter perceptual measures, and physical and physiological responses in football referees.

**Methods:**

Nine highly trained male football referees undertook two 45-minute intermittent exercise protocols in hot and humid conditions (34.2 ± 0.6°C, 62.5 ± 1.0% relative humidity). In a randomized counterbalanced crossover design, 1 of 2 beverages were given before the warm-up: a 0.01% menthol solution or a placebo noncaloric solution. Physical performance was quantified as total distance covered in each of the three 15-minute exercise blocks. Core temperature, heart rate, thermal sensation and thermal comfort were measured at rest and after each exercise block.

**Results:**

No changes were observed between trials and over time for distance covered. No main effect of mouth rinse was observed for core temperature and heart rate, but both increased over time in all conditions (*P* < 0.001). Thermal sensation and thermal comfort were significantly improved with menthol after mouth-rinsing (*P* < 0.05), but with no differences at any other time-point.

**Discussion:**

These results indicate that non-thermal cooling oral stimuli provide immediate behavioral changes but may not influence physiological or physical responses in football referees, during intermittent exercise in hot and humid environments.

**Clinical Trial Registration:**

www.clinicaltrials.gov, identifier NCT05632692.

## 1 Introduction

A competitive football match play is regulated by a referee, two assistant referees, and a sideline official. In a whole sports season, 1.3 million referees enter the football pitch every week to regulate players’ behavior and to regulate the rules of the game (1). Despite these statistics and the important role in ensuring that players and others involved maintain the laws of the game, very little scientific literature is available on football refereeing, especially when compared with players (2).

Intermittent exercise efforts (i.e., sprinting) in the heat is recognized to augment thermoregulatory strain (3), reducing physical performance (e.g., distance covered) and disturbing motivation levels (4), when compared to similar exercise in temperate conditions, concretely in football players. In fact, substantial changes to football players’ performance as a consequence of exercising in the heat are observed, with a reduced number of sprints performed by players (−10%), as well as a reduction in the distance covered at high intensity (5) and total distance covered during games (6). These changes result from anticipatory pacing to mitigate excessive increases in core and muscle temperature, as well as in thermal sensation (7, 8).

Considering that football players and football referees are allowed the same strategies to cope with the heat, it is plausible that the extreme conditions may also affect referees’ physical and physiological performance, compromising their skills to monitor and control important events during the game (9, 10). In addition, referees cover 7.5–11.5 km per match play (11, 12), and an elite football referee spends 42% of the match running at high intensity (18.1–24 km h^−1^) (13, 14). Therefore, it can be anticipated that the activity profile of elite referees is demanding and they must be considered as athletes (15). This way, it is fair that their training and performance may be given with due consideration from sport sciences, particularly regarding heat-related fatigue and strategies to cope with the heat, such as cooling interventions.

Menthol is an organic compound that incites non-thermal cooling effects on target receptors, throughout the body regions, depending on if it is applied internally or externally (16). Although menthol does not prevent heat gain or reduce core temperature, it seems to help the athlete to feel perceptually cool, being further able to perform for a longer period of time (17–19) or at a higher power output (20–22). Specifically, menthol activates the transient receptor potential cation channel subfamily M member 8 (TRPM8), which responds to cold stimuli. This activation is responsible for the cold sensation experienced from this compound and for the eventual improvements in thermal sensation, thermal comfort, rating of perceived exertion (RPE), and performance (23, 24). A recent systematic review concluded that mouth rinsing with a menthol solution (0.01%) during exercise in the heat significantly improved physical performance, mainly in continuous exercises (25). Also, considering the current body of evidence, a recent consensus statement found that research regarding the effect of menthol on intermittent exercise and on elite athletes was insufficient, while providing greater attention to endurance efforts and recreational participants (26). Adding this to the fact that menthol is simply transportable and low cost, it is worth understanding if menthol mouth rinse might be a viable non-thermic alternative to improve intermittent exercise in the heat (27).

In addition to the fact that very few studies have evaluated the ergogenic effect of menthol in intermittent exercise protocols, no study has evaluated the effects of internal cooling strategies on football referees. Accordingly, the primary purpose of the current study is to analyze the effects of a menthol solution mouth rinse on football referees’ physical performance and perceptions of the environment, during a standardized laboratory exercise protocol performed in the heat. We hypothesized that menthol mouth rinsing will improve perceptual responses and consequently increase physical performance without increasing physiological strain in football referees, while exercising in the heat.

## 2 Materials and methods

The study has been approved by the Ethics Committee of the Faculty of Nutrition and Food Sciences of the University of Porto, Portugal (Report 112/22 CEFCNAUP 2022), conducted in accordance to the Declaration of Helsinki for human studies (28) and developed considering the guidelines from Consolidated Standards of Reporting Trials (CONSORT) (29). Also, the protocol of the trial was registered on www.ClinicalTrials.gov (NCT05632692; 20 November 2022).

### 2.1 Participants

Nine healthy non-heat-acclimated highly trained (30) male football referees registered in the Portugal Football Federation participated in the study (Table 1). The participants were recommended to maintain training regimes from one trial to the following and abstain from exercise 24 h before each trial. To minimize the difference in muscle glycogen levels and respiratory exchange ratio (RER) between trials, participants were advised to consume their habitual diet and to repeat pre-exercise food intakes from one trial to the next. The participants were also asked to ingest 2–3 L of water in the day before each session.

**Table 1:** Participants characteristics.

Age (years)	33.4 ± 5.2
Height (m)	1.8 ± 0.1
Weight (kg)	73.2 ± 6.2
Body mass index (kg/m^2^)	22.4 ± 1.1
Body fat (%)	11.9 ± 3.6
Lean body mass (kg)	64.3 ± 6.0
Hours of training per week (h)	7.6 ± 2.1
Experience as a referee (years)^a^	16.7 ± 5.1
Experience as an elite referee (years)^b^	7.4 ± 2.3

Data are presented as mean ± SD (*n* = 9).

^a^ Experience as a referee considers the period (number of years) since the beginning of professional activity.

^b^ Experience as an elite referee considers the period (number of years) of professional activity in C1 or C2 elite categories.

For sample size and statistical power calculations, power analysis was based on changes in thermal sensation after the internal administration via mouth rinsing or ingestion of menthol solution. A type I error of 5% and a power of 85%, with a statistical significance (*P*-value ≤ 0.05) and a moderate effect size of 0.54 [differences in thermal sensation were considered following Jeffries and Waldron results (31)], were considered, using G*Power 3.1.9.2®. A sample size of at least eight participants was determined. Inclusion criteria required participants to be: highly trained male field football referees registered in Portuguese Football Federation, aged ≥18 and ≤45 years, with normal weight (body mass index ≥18.5 and ≤24.9 kg/m^2^), and available to participate in the familiarization session and in the two experimental sessions. Participants were excluded from the present study if they were under the influence of any medications that may affect urinary parameters, thermoregulation mechanisms, circulatory system, thyroid and pituitary function, or metabolic status; had injury, diabetes, autoimmune disease, cardiovascular disease, or obstructive disease of the gastrointestinal tract (e.g., diverticulitis, inflammatory bowel disease); were diagnosed with schizophrenia, bipolar disorder, or other psychotic disorders, as well as eating disorders; and had a magnetic resonance imaging scan scheduled within 48 h after the experimental trials (32).

### 2.2 Study design

A randomized single-blinded, counterbalanced, crossover trial with two conditions was performed. After fulfilling the eligibility criteria, and carrying one introductory meeting and a familiarization session, the participants were ascribed to two experimental days for undergoing two different randomly ordered experimental conditions. Each condition was comprised of a 45 min football protocol [intermittent Soccer Aerobic Fitness Test (SAFT-45)] (33), with the administration of one of two beverages before warm-up (pre-cooling). There was a minimum washout period of 7 days between the familiarization session and the first trial, as well as between the first and second trials, to reduce carryover effects from the previous condition and to assure an adequate exercise recovery. The trials took place in an experimental room, with temperatures ranging from 33.6 to 35.4°C and relative humidity ranging from 58.0% to 63.5%.

The main researcher assigned the allocation sequence for the order of the trial conditions to every participant, recurring to individual randomization from a computer-generated random order. The main researcher was not blinded to the type of study condition, had access to the allocation sequence list, and retrieved the randomization code prior to each experiment to prepare the beverages. All other investigators and the outcome surveyors were blinded to the study condition and group allocation, and the participants were blinded to the beverages’ composition and aim of the study.

### 2.3 Introductory meeting

The research team presented the study details to the participants, before the experimental trials. All stages of the study as well as the tools and procedures that were going to be implemented were described. After understanding and accepting to share their information, the participants signed the informed consent.

### 2.4 Familiarization session

All participants underwent a familiarization session. All experimental procedures were entirely described and tested, so that the familiarization trial was as close as possible to the experimental trials, replicating the exercise protocol.

### 2.5 Exercise protocol

The SAFT-45 is an adaptation of the original SAFT-90, consisting of sets of 15 min of predetermined intermittent football-specific protocol (34). The protocol imitates the intermittent and multi-directional nature of football match play, with regular changes in direction and activity (35). It is based on time–motion analysis data from the English Championship–level match play acquired during the 2007 season (33) and has the goal to simulate the activity demands and physiological reactions of a football game (36). Participants navigate around a 20-m agility course in an intermittent fashion via standing (0 km h^−1^), walking (5.5 km h^−1^), jogging (10.7 km h^−1^), striding (15.0 km h^−1^), or sprinting (maximal effort) (37). The protocol is divided into equivalent 15-min activity profiles, lasting 45 or 90 min, and can be performed indoors. The type of movement activity and intensity is controlled using verbal signals from an audio MP3 file (33). The participants of the present study conducted a normal pre-match routine regarding rest and nutrition, and a 5-min warm-up preceded the 45-min protocol.

### 2.6 Solution formulation

The participants were given one of two beverages: Beverage A—menthol solution was formulated by crushing non-caloric menthol lozenges into small pieces (Halls Extra Strong, Mondelez International, Birmingham, United Kingdom) weighed to obtain a concentration of 0.05% and dissolved in warm deionized water (32). After complete dissolution, the menthol solutions were diluted to a 0.01% concentration (i.e., 20 ml of the 0.05% solution were diluted in 80 ml of deionized water) (17, 18, 38); Beverage B—placebo solution was prepared using a non-caloric berry-flavored sweetener consisting of sucralose (Crystal Light, Don Mills, Ontario, Canada) (32). Prior to use, both solutions were prepared for mouth rinse and warmed at room temperature. The subjects were given, in a pre-cooling mode, 75 ml of the solution to rinse prior to the warm-up, divided into three equal parts (25 ml each). Each part was made available to the participant every 1 min (3 min of pre-cooling) and rinsed for 10 s.

### 2.7 Physiological responses

#### 2.7.1 Heart rate

Heart rate was measured through heart rate monitors (Polar H10 Heart Rate Sensor, USA) continuously throughout the exercise period and reported at baseline (before warm-up) and after each 15-min exercise block. Data were visualized and exported recurring to Polar Vantage V and Polar Flow Sync software and calculated by minutes. PolarH10 can accurately measure the mean heart rate and low-frequency oscillations (up to 0.15 Hz) of heart rate at rest and during the exercise (39).

#### 2.7.2 Core temperature

Core temperature was evaluated continuously through a telemetric pill ingested 60 min prior the start of the session (BodyCap®, Hérouville-Saint-Clair, France) and reported at baseline (before warm-up) and after each 15-min exercise block. Given the satisfactory precision, the ability to measure in field-based situations, and being non-invasive, the ingestible telemetric temperature pill was suitable to assess the core temperature during exercise in different settings (40).

#### 2.7.3 Sweating rate

Sweating rate was estimated according to the following equation (41):

$$\frac{\begin{array}{c} \mathrm{Pre}\text{-}\mathrm{exercise}\;\mathrm{body}\;\mathrm{weight}-\mathrm{post}\text{-}\mathrm{exercise}\;\mathrm{body}\;\mathrm{weight}\;\\ +\;\mathrm{fluid}\;\mathrm{intake}-\mathrm{urine}\;\mathrm{volume} \end{array}}{\mathrm{exercise}\;\mathrm{time}\;\mathrm{in}\;\mathrm{hours}}\;$$

Pre-exercise body weight was measured after participants emptied their bladder. Body weight was assessed on a digital platform scale (InBody 270, InBody CO., LTD, South Korea), with minimal clothing. Post-exercise body weight was recorded also with minimal clothing, after participants’ towel off themselves, at the end of the protocol. Urinary excretion was not considered since the protocol was continuous (no half-time) and therefore the participants did not go to the bathroom. Fluid intake was evaluated via the measurement of mass change to the nearest 0.1 ml of the individual bottles, provided to the participants, at the warm-up and collected at the end of the session (Seca, Hamburg, Germany). The research team advised the participants not to spit out the fluids at any time of the protocol. Also, fluid intake was only allowed in the 30th minute of the exercise protocol, to simulate “cooling break–water break” (32) rule implemented by FIFA. FIFA's guidelines for extreme heat conditions (i.e., wet bulb globe temperature > 32°C) refer cooling breaks to be mandatory in both halves of a match, around the 30th minute and 75th minute, so that football players and referees may rehydrate (42).

#### 2.7.4 Blood glucose and lactate levels

Blood lactate and glucose levels were assessed via a fingertip sample before and after the exercise protocol, recurring to a Blood Lactate Meter (Lactate Pro 2, Arkray, Ltd., Koka-shi, Shiga, Japan) and a Glucometer (FreeStyle Precision Neo, Abbott Laboratories, USA), respectively. Both devices have been validated previously (43, 44).

#### 2.7.5 Hydration status

Hydration status was evaluated through urine-specific gravity (USG), recurring to urine test strips (Combur10 Test M, Roche, Switzerland) and a Urisys 1100® analyzer (Roche, Switzerland) before the start and at the end of the session.

### 2.8 Perceptual measures

#### 2.8.1 RPE, thermal sensation, thermal comfort, and perceived thirst

RPE was recorded through the CR-10 Borg scale (45) from 0 (“rest”) to 10 (“maximal effort”). Thermal sensation was recorded with a 9-point scale (33) from −4 (“very cold”) to 4 (“very warm”) (46). Thermal comfort was assessed according to a 6-point scale from to −3 (“very uncomfortable) to 3 (“very comfortable”) (47). Perceived thirst was evaluated recurring to a 7-point scale from 1 (“not thirsty at all”) to 7 (“very, very thirsty”) (48).

### 2.9 Physical parameters

#### 2.9.1 Distance covered

Distance covered was calculated based on the number of shuttle run routes taken by the participants, multiplying the number of routes by the length of each route − 20 m (51). The number of shuttle run routes was measured using an app (https://simplecounter.app/). Counting was started after the warm-up, at the beginning of the 45-min exercise protocol.

### 2.10 Experimental procedure

Upon arrival at the laboratory, having abstained from vigorous exercise in the 24 h before the test, and after the telemetric pill ingestion, a urine sample was collected for a USG test to check hydration status. Both trials were schedule at the same time of the day to control for circadian variations. Body mass was assessed to gauge sweat loss, and water bottles were weighed after this (and immediately after the end of the exercise protocol).

After body mass assessment, heart rate and core temperature measurement devices were activated. Then, pre-exercise blood lactate and glucose levels evaluation occurred, through finger prick to capillary blood draws.

Next, participants were taken to the experimental room where the exercise protocol took place. Despite the short duration of the exercise protocol, internal temperature was closely monitored to ensure prevention of heat-induced illness.

When the participants arrived at the room, pre-cooling began. So, beverages (previously prepared and stored at room temperature) were distributed to the referee. Total volume of beverages for pre-cooling previously described (75 ml of Beverage A or B) (49) were divided into three equal parts and made available to the participant every 1 min. Participants were instructed to swill both beverages for 10 s before spitting into a bowl without swallowing. Perceptual measures (thermal sensation, thermal comfort, and perceived thirst) were evaluated before the beginning of the pre-cooling and after the last mouth rinse. Afterward, the participants began to warm-up, performing the same exercise protocol for 5 min. A water bottle was offered to the participants, and they were instructed to drink *ad libitum* but only in the “water break” (at the end of the second 15-min block), in order to mimic FIFA's “cooling–water” break. The temperature of the water was measured before the water break, using a digital thermometer (YSI 409B, Yellow Springs Instruments, Ohio, USA). During the warm-up, as well as after each 15-min block, environmental conditions were measured according to the Kestrel 5400 Heat Stress Tracker (Kestrel Instruments®, Boothwyn, PA, USA) (32).

**Figure 1:**
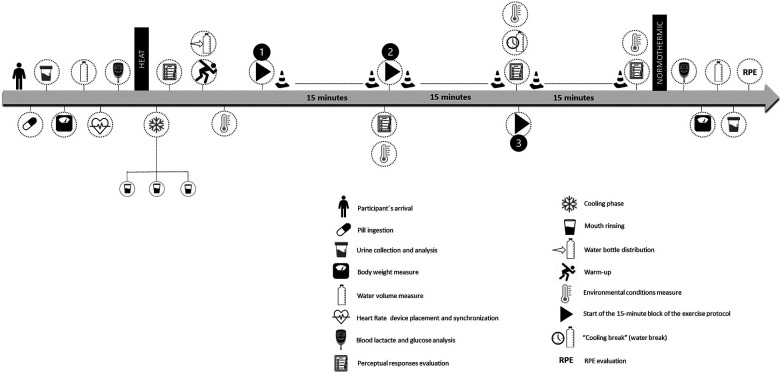
Schematic representation of the experimental trial.

The exercise protocol then started and perceptual measures were evaluated at the end of each 15-min block. At the end, under normothermic conditions, blood lactate and glucose levels were measured again and afterward, the RPE scale was applied to the participants. Figure 1 provides an overview of the measurements and the study protocol.

### 2.11 Statistical analysis

All statistical analyses were performed using SPSS (IBM SPSS Statistics 28 Inc., USA). Statistical significance was accepted at *P* < 0.05. Normal distribution was tested using the Shapiro–Wilk test. Data are presented as mean ± SD, unless otherwise indicated. Single time point data were examined for within-group effects across conditions using a one-way repeated-measures analysis of variance (ANOVA). A two-way repeated-measures ANOVA was used to test for within-group effects across time in both conditions. If sphericity was violated, a Greenhouse–Geisser correction was applied. When a significant difference was found for main effects (trial or time), *post-hoc* pairwise comparisons were made incorporating a Bonferroni adjustment. The magnitude of effect was calculated with partial eta-squared $\left(\eta_{p}^{2}\right)$ according to the following criteria: 0.02, small difference; 0.13, moderate difference; and 0.26, large difference (49). A paired *t*-test was used to compare single parameter differences and magnitude of effect calculated (Cohen's *d*) according to the following criteria: 0.2, small difference; 0.5, moderate difference; and 0.8, large difference (49).

## 3 Results

### 3.1 Trial conditions and pre-exercise biochemical measures

The menthol and placebo trials did not differ for environmental conditions (menthol: temperature 34.2°C ± 0.6°C, relative humidity 62.5% ± 1.0%, wet bulb globe temperature 30.3 ± 0.5°C; placebo: temperature 34.7°C ± 0.7°C, relative humidity 60.4% ± 2.4%, wet bulb globe temperature 30.4 ± 0.4°C; *P* ≥ 0.05), or pre-trial USG levels (menthol: 1.013 ± 0.01 USG, placebo: 1.012 ± 0.01 USG, *P* = 0.347). Also, no differences were detected for pre-trial glucose and lactate levels (menthol: glucose 97.7 ± 19.2 mg/dl, placebo: 93.1 ± 10.2 mg/dl, *P* = 0.406; menthol: lactate 2.48 ± 0.62 mmol/L, placebo: 1.90 ± 0.61 mmol/L, *P* = 0.111) (Table 2).

**Table 2:** Biochemical measures before and after exercise, across menthol and placebo trials.

Parameter	Pre-test (*n* = 9)			Post-test (*n* = 9)		*P*-value
	Menthol	Placebo	*P*-value	Menthol	Placebo	
USG	1.013 ± 0.01	1.012 ± 0.01	0.347	1.013 ± 0.05	1.012 ± 0.05	0.282
Blood glucose (mg/dl)	97.6 ± 19.6	93.1 ± 10.2	0.406	99.2 ± 12.5	92.7 ± 10.2	0.119
Blood lactate (mmol/L)	2.5 ± 0.6	1.9 ± 0.6	0.111	2.7 ± 0.5	2.1 ± 0.4	0.321

Data are presented as mean ± SD (*n* = 9).

### 3.2 Physiological responses to mouth rinse

**Figure 2:**
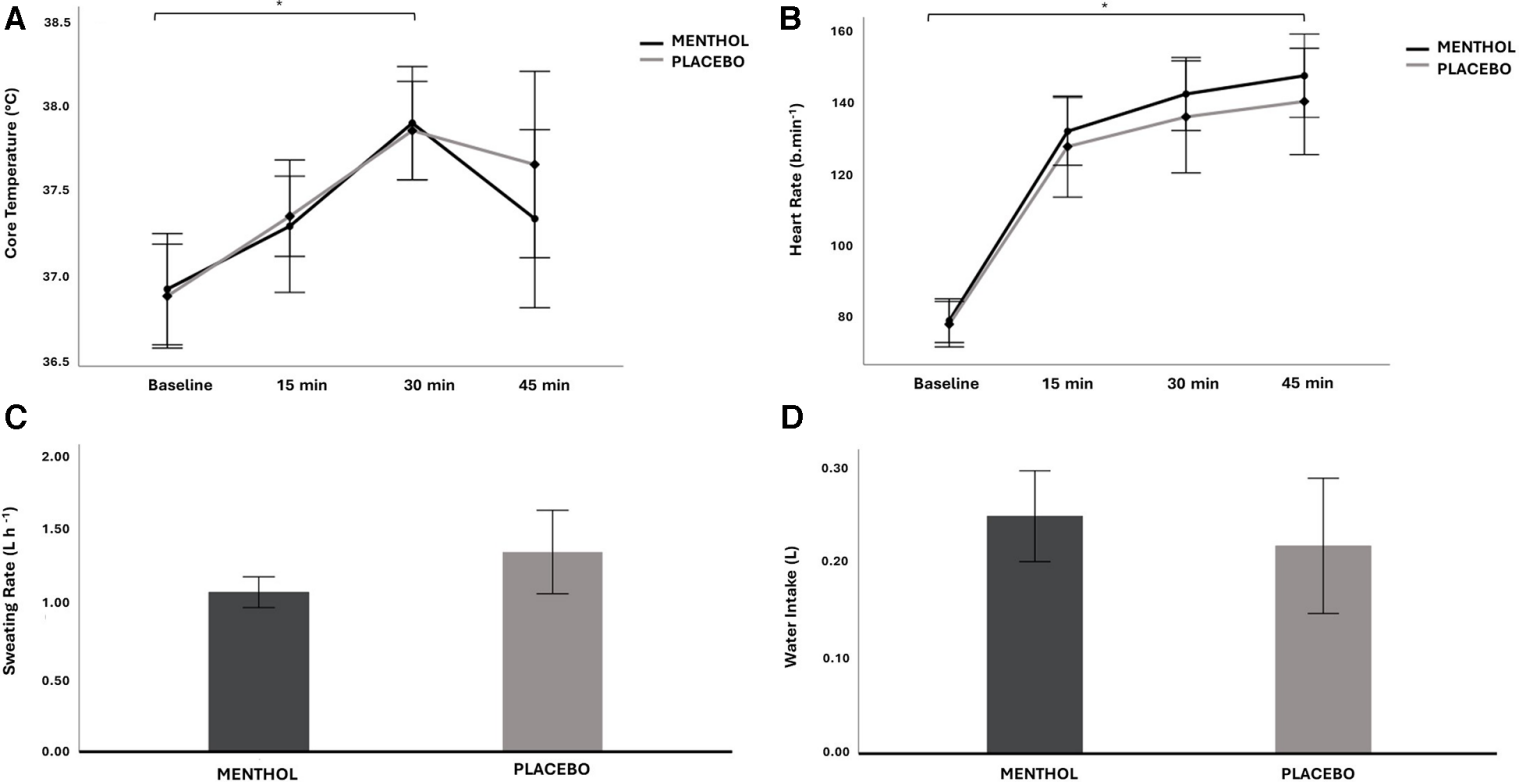
Mean ± 95% CI for core temperature (**A**), heart rate (**B**), sweating rate (**C**) and water intake (**D**) across the menthol and placebo conditions, for all time points. *Significant difference between exercise blocks, with no difference between groups.

There was no main effect of mouth rinse on core temperature, heart rate, sweating rate, post-exercise hydration status, and *ad libitum* water intake, as well as an interaction effect between mouth rinse and time on core temperature and heart rate (core temperature: menthol: *F*_(1,8)_ = 0.26, *P* = 0.622, $\eta_{p}^{2}=0.032$, menthol × time: *F*_(3,24)_ = 1.1, *P* = 0.290, $\eta_{p}^{2}=0.441$; heart rate: menthol: *F*_(1,8)_ = 2.64, *P* = 0.143, $\eta_{p}^{2}$  0.248, menthol × time: *F*_(3,24)_ = 1.83, *P* = 0.212, $\eta_{p}^{2}=0.186$; sweating rate: menthol: *F*_(1,8)_ = 1.61, *P* = 0.240, $\eta_{p}^{2}=0.168$; hydration status: menthol: *F*_(1,8)_ = 1.33, *P* = 0.282, $\eta_{p}^{2}=0.143$; water intake: menthol: *F*_(1,8)_ = 0.13, *P* = 0.724, $\eta_{p}^{2}=0.016$). Blood lactate and glucose levels at the end were also not significantly different between conditions (lactate: *F*_(1,8)_ = 2.91, *P* = 0.321, $\eta_{p}^{2}=0.619$; glucose: *F*_(1,8)_ = 3.02, *P* = 0.119, $\eta_{p}^{2}=0.276$). Physiological responses are presented in Table 2 and Figure 2.

Core temperature (°C) and heart rate (b min^−1^) significantly increased over time (core temperature: *F*_(15.75,0.33) _= 17.34, *P* < 0.001, $\eta_{p}^{2}=0.979$; heart rate: *F*_(1.268,10.143)_* *= 151.06, *P* < 0.001, $\eta_{p}^{2}=0.950$). Heart rate was higher at every time point than the previous time point (all *P* < 0.01, all *d* > 1.1), as well as core temperature (all *P* < 0.01, all *d *> 0.9), except for the last 15-min block (*P *= 0.073, *d* = 0.18), where core temperature decreased for both conditions (Figures 2A,B).

### 3.3 Perceptual responses to mouth rinse

There was no main effect of mouth rinse on thermal sensation, thermal comfort, and perceived thirst (thermal sensation: *F*_(1,8)_ = 1.22, *P* = 0.302, $\eta_{p}^{2}=0.132$; thermal comfort: *F*_(1,8)_ = 1.07, *P* = 0.332, $\eta_{p}^{2}=0.118$; perceived thirst: *F*_(1,8)_ = 0.42, *P* = 0.537, $\eta_{p}^{2}=0.049$).

However, there was an interaction effect between mouth rinse and time on thermal sensation and thermal comfort but not on perceived thirst (thermal sensation: *F*_(4,32)_ = 9.51, *P *= 0.037, $\eta_{p}^{2}=0.135$; thermal comfort: *F*_(4,32)_ = 8.70, *P* = 0.041, $\eta_{p}^{2}=0.126$; perceived thirst: *F*_(4,32)_ = 2.95, *P* = 0.089, $\eta_{p}^{2}=0.021$). Pairwise analysis confirmed that thermal sensation was significantly lower in menthol trial after cooling (*P* = 0.022, *d* = 0.41) and thermal comfort was significantly higher also after cooling in the menthol condition (*P* = 0.044, *d* = 0.37). Thermal sensation and thermal comfort did not differ between menthol and placebo conditions at baseline and at the first, second, and third exercise blocks (all *P* ≥ 0.05).

**Figure 3:**
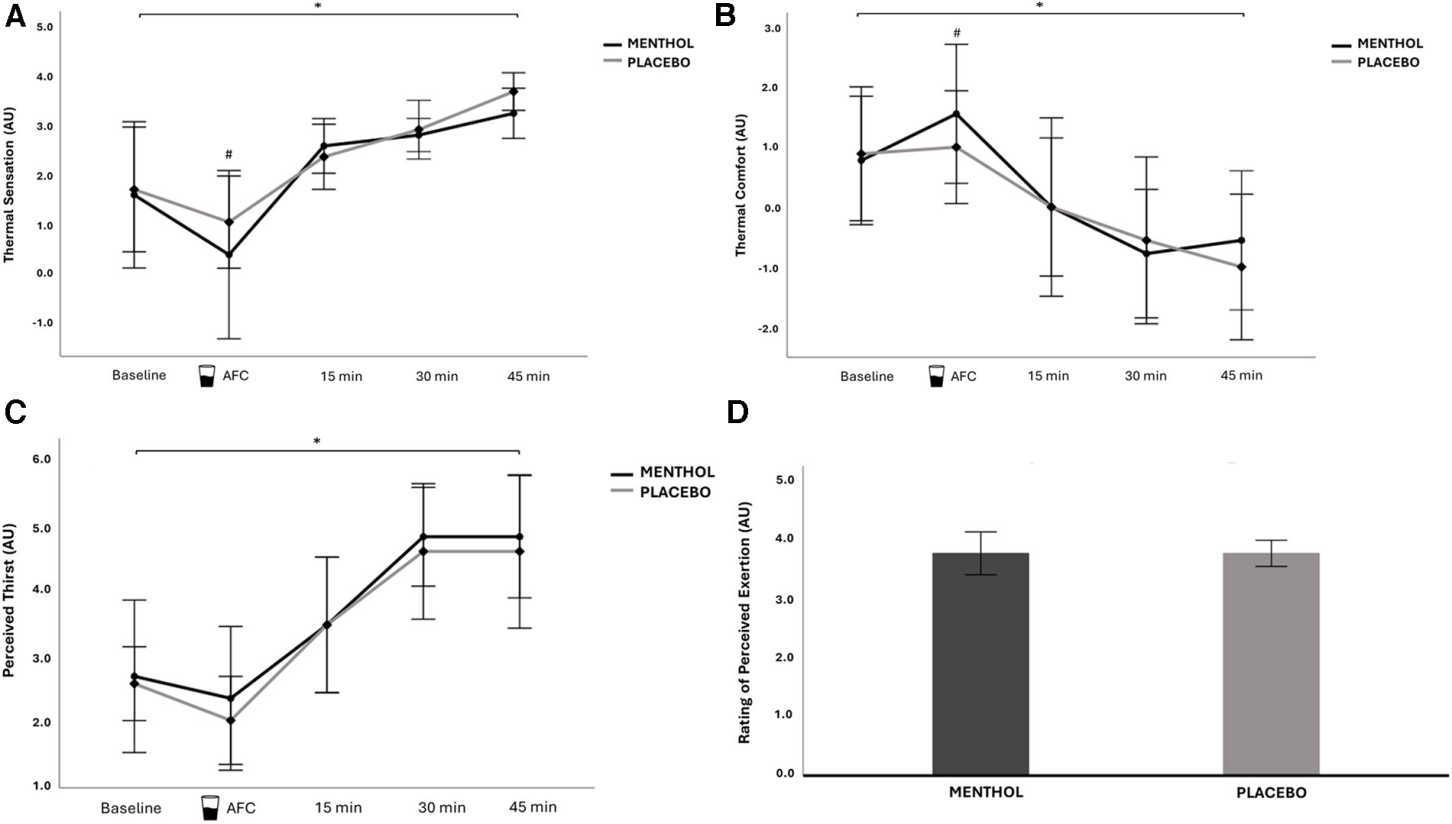
Mean ± 95% CI for thermal sensation (**A**), thermal comfort (**B**), perceived thirst (**C**) and RPE (**D**) across the menthol and placebo conditions, for all time points. *Significant difference between exercise blocks, with no difference between conditions. #Significant difference between conditions. AFC, after cooling; AU, arbitrary units.

Thermal sensation, thermal comfort, and perceived thirst significantly changed through each time point (thermal sensation: *F*_(1.27,10.215)_ = 14.10, *P* = 0.002, $\eta_{p}^{2}=0.638$; thermal comfort: *F*_(1.35,10.603)_ = 12.20, *P* = 0.003, $\eta_{p}^{2}=0.604$; perceived thirst: *F*_(2.255,18.043)_ = 29.38, *P* < 0.001, $\eta_{p}^{2}=0.786$). Thermal sensation significantly decreased from baseline to after cooling and then significantly increased over time (all *P* < 0.05, all *d* > 0.8). Thermal comfort significantly increased from baseline to after cooling and significantly decreased over time (all *P* < 0.05, all *d* > 0.6), except for the last exercise block, where thermal comfort was not significantly higher than the previous block (*P* = 1.00, *d* = 0.1). Perceived thirst significantly increased from baseline and after cooling to the second and third exercise blocks, as well as from the first to second and third exercise blocks (all *P* < 0.05, all *d* > 0.4), with no differences between any other time points. Perceptual responses are presented in Figures 3A–C.

Finally, there was no main effect of mouth rinse on RPE [*F*_(1,8)_ = 0.0, *P* = 1.00, $\eta_{p}^{2}=0.000$], as a matter of fact mean RPE in the menthol condition was equal to the placebo (*P* = 1.00) (Figure 3D).

### 3.4 Effect of mouth rinse on exercise performance

**Figure 4:**
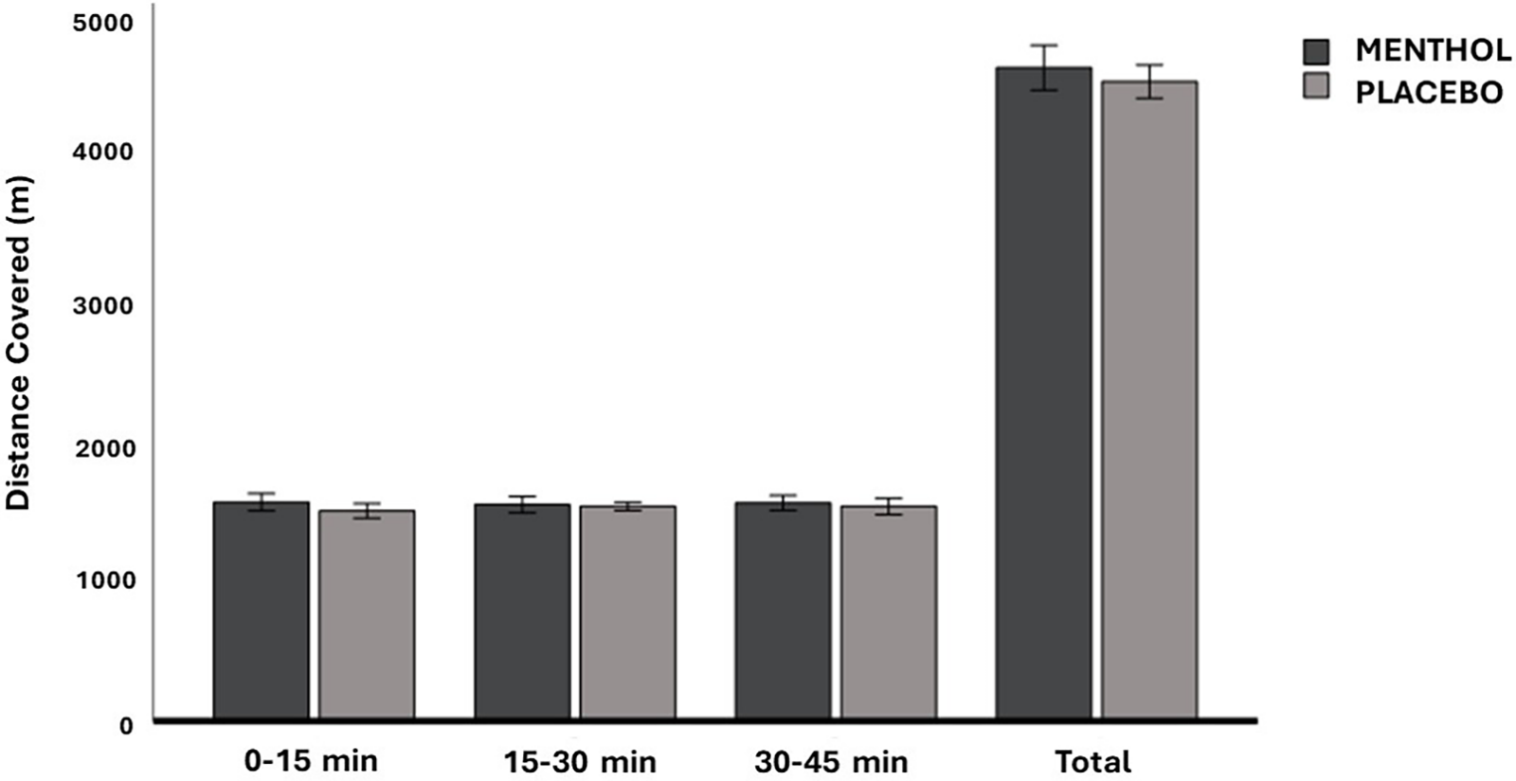
Mean ± 95% CI of the distance covered (in each exercise block and total) across the menthol and placebo conditions.

There was no main effect of mouth rinse, or interaction effect between mouth rinse and time on distance covered (Figure 4) (menthol: *F*_(1,8)_ = 1.24, *P* = 0.298, $\eta_{p}^{2}=0.134$; menthol × time: *F*_(2,16)_ = 0.23, *P* = 0.801, $\eta_{p}^{2}=0.027$). The distance covered during each of the 15-min exercise block was not significantly different between the three blocks [*F*_(2,16) _= 0.16, *P* = 0.855, $\eta_{p}^{2}=0.019$] in both conditions.

## 4 Discussion and implications

The purpose of the present study was to evaluate the effect of pre-cooling with menthol solution mouth rinse on perceptual measures and distance covered in male football referees, while performing an exercise protocol that mimics a football game. Our main findings revealed that mouth rinsing with a menthol solution ameliorated perceptual measures, enhancing thermal sensation and thermal comfort immediately after rinsing, but with no long-term effect. Also, no changes were observed in physical performance, not even in the first exercise block, refuting our initial hypothesis that an eventual improvement in the perceptual measures would benefit physical performance.

### 4.1 Effect of mouth rinse on exercise performance

Physical performance measured by the distance covered did not significantly differ between conditions, which is in accordance with previous findings that revealed no improvements in intermittent exercise performance with oral menthol, even when thermal comfort was higher, as occurred in our study (50, 51). This may be due to the fact that exercise performed in an intermittent intensity mode, interspersing high-intensity bouts with periods of rest, causes less heat stress than the same amount of continuous exercise, where stored heat is greater and core temperature and thermal sensation rise faster (52–54). We may also speculate that, despite the observed increases in heart rate and core temperature along our exercise protocol, the exercise task may have not increased over time, since the distance covered in each block was similar, with no deterioration in performance over time. This may explain the fact that menthol did not have a significant impact on physical performance in our study, which could have been detected with a more demanding exercise protocol. In addition, it was previously observed that a cooling intervention using ice slurry reduced core temperature and heart rate at the end of an intermittent exercise protocol performed in hot conditions, which was translated to an increased peak power (+4%) and work done (+2%) in the intervention group (55, 56). These performance data, combined with no differences in RPE or thermal sensation between cooling and control trials, suggested that the performance ability during intermittent sprints in the heat was more closely related to physiological responses, rather than perceived temperature. This highlights an explanation for the unchanged intermittent exercise performance in the present study, despite improved thermal sensation and thermal comfort, as a non-thermal cooling method was used. Other study showed that peak power only improved in heat (+2%) when the intervention induced reductions in core temperature, heart rate, and thermal sensation (24). For that reason, thermal internal cooling techniques may best suit intermittent exercise protocols. These findings indicates that physiological intervention might be worthy for intermittent sprinting in the heat, while physiological and perceptual manipulations should be discussed for continuous endurance exercise in heat (25).

Furthermore, even though we observed a significant increase in thermal sensation in both conditions during our exercise protocol, thermal sensation points were higher during exercise in other trials with continuous endurance efforts (18, 21, 38, 57). This also may explain why we did not find a performance improvement with oral menthol, since the “thermal stress” felt by the participants may not have been high enough to benefit from non-thermal cooling.

### 4.2 Physiological responses to mouth rinse

Consistent with the “non-thermal” mechanistic basis of menthol's cooling effects (31), there were no changes in core temperature or heart rate between conditions. Actually, our work showed lower core temperature values (menthol: 37.6 ± 0.4°C), compared to what was achieved (∼39.5°C) in other studies, where a continuous endurance exercise protocol was performed (19, 57, 58). Oral menthol significantly improved physical performance in those studies, which may be due to the higher heat storage observed. It is unlikely that the smaller increase in core temperature found in our protocol is due to atmospheric conditions, as other studies carried out under similar atmospheric conditions have higher core temperature values (18, 22, 27). Perhaps, exercise intensities achieved in our study were not sufficiently high, which is supported by the lower heart rate values (menthol: 135.5 ± 11.1 b min^−1^), compared to other findings (19, 21, 22), as well as an average RPE of 4 points, corresponding to “somewhat hard.”

It is still important to point out that core temperature increased throughout the exercise protocol but decreased in the last block possibly as a consequence of the “water break” (Figure 2A). It is expected that the increase in core temperature will be attenuated when participants hydrate themselves with sufficient fluid volumes, even if the water was at room temperature (59), as was the case.

Regarding water intake, previous studies demonstrated a larger consumption when the fluid was cold (≤5°C) compared to a control (16–19°C) (24, 60). In fact, we used a beverage temperature similar to deep body temperature to minimize the influence of visceral temperature modulation, but other studies used cooler mouth rinses (57, 61). However, as menthol exerts its cooling effect by activating subsequent stimuli (inspired air, water consumed) feel cool (17), water may be understood as cooler in the menthol trial, which may have contributed to increase the volume ingested (even though not significant).

### 4.3 Perceptual responses to mouth rinse

Menthol, by stimulating the trigeminal system, seems to directly activate reward centers in the brain to increase “central drive” and enhance work capacity (62). Activation of these areas in the brain, such as the insula/frontal operculum, the orbitofrontal cortex, and the striatum, may lower perceived exertion (63) and help improve motivation during exercise performance (64). Nonetheless, in our study, there were no significant differences in RPE between conditions. To the best of our knowledge, none of the studies that applied oral menthol in intermittent exercise found improvements in RPE. In those studies (50, 51), core temperature and heart rate values were similar to those observed in our work, supporting that menthol may not affect RPE in this type of exercise protocols, possibly due to a less physically demanding exercise task.

Our findings revealed an improvement in thermal sensation and thermal comfort after cooling, but not in subsequent time points. Another study where an intermittent exercise protocol with similar duration was performed, thermal sensation and thermal comfort were also immediately improved after menthol mouth rinse (50). However, this improvement continued throughout the exercise because oral menthol was administered every 10 min. It is highly likely that multiple moments of per-cooling during our exercise protocol would have led to an extension of the improvement in the perceptual measures. Anyhow, we intended to make our protocol as realistic as possible, getting closer to the cooling and hydration timings that exist in a football game.

Despite being expected to find differences between conditions in perceived thirst (65), it was not lower in the menthol trial. Oral menthol rising is presumed to increase the drive to breathe and ventilation, and to decrease thirst, as well as promote sensations of coolness and freshness (23, 66). Menthol stimulates oral cold receptors and may subsequently have the same effects on thirst and the hedonic process as cold water (67). As perceived thirst was not lower in the menthol trial, perhaps a greater concentration of menthol or a higher frequency of mouth rinses during the exercise may be necessary.

The findings of the current study suggest that menthol stimulation of the TRPM8 ion channel enhanced thermal comfort and sensation following mouth rinsing, suggesting that our participants were more “perceptually tolerant” to physiological heat stress. The improvement in perceptual measures right after rinsing would be expected as it was found in previous research (68). However, improvements in perception did not extend throughout the exercise, possibly indicating that a higher frequency of menthol mouth rinsing should have been experimented. This, along with a higher demanding exercise protocol, could have contributed to a possible ergogenic action of menthol. Future research should explore the influence of menthol mouth rinsing also in the advanced stages of exercise, when fatigue is traditionally high, concretely in long-lasting moderate-to-high-intensity exercise protocols.

### 4.4 Limitations

As there is no validated exercise protocol that mimics the activity of football referees, the SAFT-45 was chosen, although it has only been validated for football players. The exercise protocol was performed in an experimental room with hot and humid environments to simulate conditions of high temperature and humidity, while still ensuring stability between measures. The closed task of SAFT-45 possibly provokes an experimental artifact, because participants are not able to sprint and perform freely (either in frequency or duration). Future work should consider the benefits of a “free” task (e.g., devising a protocol with an undefined sprint duration), or participant-regulated sprint frequency, where participants could pace independently in response to non-thermal cooling interventions in the heat. This approach may enable different responses due to the elevation in pacing associated with these tasks, differentiating it from the SAFT-45, which applies a fixed duration and fixed frequency of sprinting. In this study, we opted to shorten the original SAFT-90 and used the SAFT-45, to increase adherence and ensure the crossover design with the second visit of the participants. Conversely, this may have been a major limitation of the study, because the exercise protocol lasts only 45 min and, therefore, only replicates the first half of a football match. Future work should extend the task duration to more closely replicate the team sport of interest (69), in high-level team sport players or referees.

Another possible limitation of our work was the non-use of menthol crystals to prepare the solution, as in other studies (16, 19, 20, 22). However, Kalantzis et al. showed that menthol significantly changes thermal sensory thresholds in the oral cavity (68) when the participants were asked to suck a lozenge (Halls Extra Strong menthols, Mondelez International, Birmingham, United Kingdom) immediately before repeating the measurement of the thermal thresholds on the right dorsal surface of the tongue, indicating that this substrate can also be used to trigger changes in thermal perception, being much more practical than preparing the beverage with menthol crystals, which requires a lot of time in advance.

Finally, although with the aim of understanding whether menthol would influence the perception of thirst and, consequently, the amount of water ingested, not fixing the volume of water that the referees drank during the exercise in both trials could have conditioned the perceptual results observed in the last block of exercise. However, this was not observed, since no significant differences were found for water intake and no statistical effects were observed between conditions for perceptual measurements after the 30th-min water break.

## 5 Conclusions

Mouth rinsing with a menthol solution improved thermal sensation and comfort immediately after the administration but had no long-term effect during a 45-min intermittent exercise protocol, in heat, in male football referees. In opposition of what was hypothesized, no changes were also detected in the physical performance or physiological responses. Pre-cooling with oral menthol in a hot and humid environment may promote an immediate change on perceptual measures, but not enough to improve intermittent exercise performance in football referees.

## Data Availability

The raw data supporting the conclusions of this article will be made available by the authors, without undue reservation.
